# The Involvement of the Banana F-Box Protein MaEBF1 in Regulating Chilling-Inhibited Starch Degradation through Interaction with a MaNAC67-Like Protein

**DOI:** 10.3390/biom9100552

**Published:** 2019-09-30

**Authors:** Zunyang Song, Jiajia Qin, Qiuli Zheng, Xiaochun Ding, Weixin Chen, Wangjin Lu, Xueping Li, Xiaoyang Zhu

**Affiliations:** State Key Laboratory for Conservation and Utilization of Subtropical Agro-Bioresources/Guangdong Provincial Key Laboratory of Postharvest Science of Fruits and Vegetables, College of Horticulture, South China Agricultural University, Guangzhou 510642, China; songzunyang@163.com (Z.S.); jia13424450489@163.com (J.Q.); zhengqiuli0102@163.com (Q.Z.); dingxiaochun111@163.com (X.D.); wxchen@scau.edu.cn (W.C.); wjlu@scau.edu.cn (W.L.)

**Keywords:** Fenjiao banana, ripening disorder, starch degradation, chilling injury, MaEBF1, MaNAC67-like

## Abstract

Low-temperature storage is a common strategy for preserving and transporting vegetables and fruits. However, many fruits are hypersensitive to chilling injury, including bananas. In the present study, storage conditions of 11 °C delayed the ripening of Fenjiao (*Musa* ABB Pisang Awak) banana, and the pulp could be softened after ethephon treatment. Storage conditions of 7 °C prevented fruit from fully softening, and fruit contained a significantly higher starch content and lower soluble sugar content. *MaEBF1*, a critical gene component in the ethylene signaling pathway, was repressed during ripening after fruit had been stored for 12 days at 7 °C. The expression of a series of starch degradation-related genes and a *MaNAC67-like* gene were also severely repressed. Both *MaEBF1* and *MaNAC67*-like genes were ethylene-inducible and localized in the nucleus. MaNAC67-like protein was able to physically bind to the promoter of genes associated with starch degradation, including *MaBAM6*, *MaSEX4*, and *MaMEX1*. Yeast two-hybrid, GST-pull down, and BiFC assays showed that MaEBF1 interacted with the MaNAC67-like protein, and their interaction further activated the promoters of *MaBAM6* and *MaSEX4*. The current study indicates that MaNAC67-like is a direct regulator of starch degradation and potential for involvement in regulating chilling-inhibited starch degradation by interacting with the ethylene signaling components in banana fruit. The present work paves the way for further functional analysis of *MaEBF1* and *MaNAC67-like* in banana, which will be useful for understanding the regulation of banana starch metabolism and fruit ripening.

## 1. Introduction

As the second largest fruit crop in the world, banana is an important food crops in many areas, especially for the tropical and sub-tropical regions [1]. Banana is a typical climacteric fruit that once harvested, ripens quickly and has a short shelf life. This dramatically shortens the period bananas can be stored and transported [2]. Various postharvest technologies have been employed on banana and studies to prolong the storage and shelf life have been conducted [3,4]. One of the most common methods for preserving fruit is low-temperature storage, which can be employed to prolong the shelf life of various fruit, including non-climacteric and climacteric fruits [5,6,7], such as banana [8]. However, banana fruit is hypersensitive to low temperatures, with chilling injury (CI) easily induced when fruit are subjected to temperatures below 11 °C. Exposure to temperatures this low can result in cellular membrane damage, flavor loss, and abnormal fruit ripening [9]. Several previous studies have also reported that CI significantly repressed starch degradation in banana [10], as well as potato [11] and melon [12], but the underlying mechanism for abnormal fruit ripening remains unclear.

CI in banana fruit seems to be closely associated with a decrease in ethylene binding ability, resulting in abnormal fruit ripening [13]. Ethylene synthesis and signaling have been widely studied in the *Arabidopsis* plant, where the expression of genes associated with the ethylene signaling pathway, downstream and feedback regulation was stimulated by ethylene receptors [14]. Two F-box proteins, EIN3 binding F-box-1/2 (EBF1/2), play a pivotal role in ethylene signaling transduction, which shows an increased expression level after exogenous ethylene application [15]. EIN3 can be degraded by EBF1/2 through an ubiquitin/proteasome-mediated pathway under no ethylene condition [16]. The function characterization of *EBF* genes has been studied in just a few fruits, including tomato [17,18], apple [19], and papaya [20]. At the onset of fruiting, the gene expression of *SlEBF1/2* has been shown to increase sharply, suggesting that *SlEBF* genes may be pivotal components in regulating ethylene signal responses during tomato fruit ripening. Furthermore, the senescence and ripening of tomato were accelerated by silencing the *SlEBF1/2* gene [18]. Tomato fruits that were over-expressing *SlEBF2-like* had an elongated fruit shape and a postponed fruit ripening and development phenotype [17]. In apple, the expression of *EBF1/2* increased sharply during fruit ripening and EBF1 repressed the promoter transactivation of *PG1* gene by regulating the activity of EIL1/2/3 [19]. Although the function of EBF in ethylene response has already been investigated in model plants, the roles of EBF in the ripening of some fruit of great economic importance, such as banana, are not clear.

NAC (NAM, ATAF1/2 and CUC2) is one of the most important transcription factor (TF) families in plants. NAC proteins share a conserved DNA-binding domain and a diversified transcriptional activation domain at the N-terminus and C-terminus, respectively [21,22]. Numerous NAC TFs have been identified and characterized in different plants species, and studies have showed that NAC proteins participate in a variety of developmental stages, plant senescence, and biotic and abiotic stress responses [23,24,25]. Recently, a growing number of studies have worked on the roles of NAC proteins in fruit ripening. MaNAC1/MaNAC2 have been found to participate in the banana fruit ripening via interacting with ethylene signaling component MaEIL5 [26]. In loquat fruit, EjNAC1 activated the expression of genes related to lignin biosynthesis [27]. In papaya, CpNAC1 positively regulated carotenoid biosynthesis during fruit ripening via activating the expression of *CpPDS2/4* [28]. In tomato, SNAC4- and SNAC9-regulated genes involved in ethylene synthesis positively regulated the fruit ripening process [29,30]. Additionally, NAC–NOR mutations attenuated multiple metabolic processes in tomato, prolonging the fruit shelf life [31].

Fenjiao (*Musa* ABB Pisang Awak) is a popular banana cultivar with good flavor and high resistance to abiotic stress, and is widely consumed and cultivated in China [32]. However, Fenjiao fruit have a short shelf life compared with other commercial cultivars [33]. Previous studies have indicated that the softening of banana fruit is related to the degradation of starch [34]. However, the involvement of NAC TF in starch degradation associated with fruit quality during low-temperature storage and ripening has not been well understood. The aims of this study were to understand fruit softening profiles after chilling injury, and reveal the possible molecular mechanism of chilling-induced abnormal ripening. The effect of chilling temperature storage (7 °C, CTS) on starch metabolism and sugar conversion during both the storage and ripening periods of banana fruit were studied. The roles of MaEBF1 and MaNAC67-like in ethylene signaling, fruit ripening, and starch degradation were investigated, to better understand fruit chilling tolerance and to improve fruit shelf life.

## 2. Materials and Methods

### 2.1. Plant Materials and Treatments

Fenjiao banana fruit (*Musa* ABB Pisang Awak cv. ‘Guangfen No.1’) at 85–90% maturation were collected from a local orchard in Nansha, which is close to Guangzhou, China. The fruit were manually separated into individual fingers, and fruit with uniform shape, weight, maturity, and without visual defects were selected. The fruit was first dipped in a 0.2% (w/v) hypochloride solution for 10 min and then were soaked in 500 μL·L^−1^ mixture of iprodione (Kuaida, Jiangsu, China) and prochloraz (Huifeng, Jiangsu, China) for 1 min. Fruits were under air dry at 25 °C for 2 h and treated as follows.

The selected fruits were divided into three groups of 200 fingers randomly. Each treatment included 20 sub-groups with 10 fingers of banana each, which were placed in unsealed plastic bags (0.02 mm in thickness). Samples were collected on the basis of color index, chilling index, and fruit firmness change during ripening. For the control group, fruits were stored directly at 25 °C with 90% relative humidity for 12 days; samples were collected at 0, 3, 6, 9, and 12 days until the fruit had fully ripened. For the 7 °C and 11 °C treatments, fruit were stored in chambers (MIR-554-PC, Ehime Prefecture, Japan) at 7 °C and 11 °C, respectively. After 6 and 12 days of storage, banana fruit were treated with 1000 μL·L^−1^ of ethephon for 1 min, and then placed in a room at 25 °C with 90% relative humidity to ripen. Samples were collected at 0, 3, 6, 9, and 12 days during cold storage, and 1, 3, 5, and 7 days after ethephon treatment. All samples were frozen in liquid nitrogen after sampling and immediately stored at −80 °C. All of the treatments were conducted with three biological replicates.

### 2.2. Fruit Ripening and Chilling Injury Index Assessment

Fruit firmness and the color index were measured as described by Zhu et al. [3]. CI of Fenjiao bananas was evaluated by visual browning of the skin: (0) No chilling injury at all; (1) less than 5% of skin area; (2) 5–25% of skin area; (3) 25–50% of skin area; (4) 50–75% of skin area; and (5) more than 75% of skin area [35].

### 2.3. Starch, Fructose, Glucose, and Sucrose Content

The total starch and fructose in the Fenjiao banana pulp was measured using a Starch and Fructose Assay Kit (Sigma-Aldrich Shanghai Trading Co. Ltd., Shanghai, China), according to the manufacturer’s instruction. The glucose and sucrose content was quantified enzymatically according to the protocol described by Xiao et al. [36].

### 2.4. Fruit Cell Structure Observations

The microstructure of banana sarcocarp cells was evaluated using transmission electron microscopy (TEM) as previously described [20,37].

### 2.5. Gene Expression Profiles Analysis

The total RNA was isolated using an RNA Extraction Kit (Aidlab Biotechnologies Co., Ltd., Beijing, China) from fruit pulp samples, and then reverse transcribed to first strand cDNA. RT-qPCR was used for gene expression analysis and performed as previously described by Song et al. [38]. Primers used in this experiment are listed in Table S1. Gene expression level was calculated using the method of 2^−ΔΔCt^ [39]. Actin was selected as internal reference gene for banana, as validated by Chen et al. [40]. Three independent biological replicates were measured for the analysis.

### 2.6. Construction of a cDNA Library

The cDNA library was constructed as previously described by Ding et al. [20]. The total mRNA was isolated by an RNA Extraction Kit (Aidlab Biotechnologies Co., Ltd., Beijing, China). The cDNA library was constructed using mixed RNA samples of fruit stored at 7 °C (1, 2, 3, 6, 9, 12 days) by the SMART™ cDNA Library Construction Kit (Clontech, Cat. No. 634901, Fitchburg, WI, USA). The yeast transformation was done using a Yeast Maker Transformation System 2 Kit (Clontech, Cat. NO. 630439). Yeast two-hybrid (Y2H) assay (Clontech, Cat. No. 630489) was conducted according to the manufacturer’s instructions. Yeast plasmid was extracted using an Easy Yeast Plasmid Isolation Kit (Clontech, Cat. No. 630467).

### 2.7. Y2H Assay

The Y2H screening was conducted using the Matchmaker Gold Yeast Two-Hybrid System (Clontech, Cat. No. 630489). The full coding region sequence (CDS) of MaEBF1 was ligated to a pGBKT7 vector. The toxicity testing and self-activation of full-length MaEBF1 protein indicated that MaEBF1 did not cause toxic effects (data not shown) and had no self-activation in the yeast host cell. The MaEBF1 protein was used as the bait protein to screen the candidate interacting proteins in the cDNA library. A MaNAC67-like protein was screened as the interacting protein for MaEBF1. Next, the full lengths of MaNAC67-like CDS were cloned separately into pGBKT7 and pGADT7 vectors. All the primers used in the assay are listed in Table S1. The yeast strains were co-transformed with MaEBF1 + MaNAC67-like to confirm their interaction.

### 2.8. Bimolecular Florescence Complementation (BiFC) Assay

The full-length of MaEBF1 and MaNAC67-like without their stop codons were ligated into the pBIFC vectors using Gateway technology. BiFC experiment was performed as previously described by Ding et al. [20]. All the primers used in present assay are presented in Table S1.

### 2.9. GST Pull-Down Assay

The full CDS of MaEBF1 was inserted in PET-GST and MaNAC67-like cloned to PET-28a vector. The fused vector was transformed into BM Rosetta (DE3) cells and induced with 0.1 mM IPTG. MaEBF1-GST and MaNAC67-like-His were incubated at 20 °C for 6 h and the unpurified proteins were obtained from MaEBF1 and MaNAC67-like. The MaEBF1-GST protein was purified using the GST Purification Kit (Clontech, Cat. No. 635619, Fitchburg, WI, USA) and the MaNAC67-like-His protein was purified using the Ni-NTA His Purification Kit (Clontech, Cat. No. 635658). The purified proteins were analyzed firstly with anti-His antibody and anti-GST antibody (Sigma-Aldrich), and then with secondary mouse anti-rabbit IgG peroxidase antibody (Thermo Scientific, Waltham, MA, USA) by western-blot. Primers used in the experiment are listed in Table S1.

### 2.10. Subcellular Location Analysis

The full CDS of MaEBF1 and MaNAC67-like without stop codons were ligated into a pENTR/D vector (Invitrogen, Waltham, MA, USA). MaEBF1-GFP and MaNAC67-like-GFP fusion proteins were produced using the LR clonase enzyme (Invitrogen, USA). The resulting plasmids and control GFP vector were then transformed in the *Agrobacterium tumefaciens* strain GV3101. Subcellular location analysis in detail was described in previous work by Ding et al. [20].

### 2.11. Dual-Luciferase Transient Expression Assays

Banana DNA was extracted using the DNeasy Plant Mini Kit (Tiangen, Beijing, China). The promoters of 11 starch degradation-related genes including *MaBAM3/MaBAM4/MaBAM6/MaBAM7/MaBAM8/MaPWD1/MaGWD1/MaAMY3/MaISA2/MaSEX4/MaMEX1* were isolated from the banana genome (ftp://ftp.jgi-psf.org/pub/compgen/phytozome/v9.0/C banana) and cloned using the primers presented in Table S1.

The promoters of the starch degradation-related genes were cloned into the pGreenII 0800-LUC double-reporter vector, and MaEBF1 and MaNAC67-like were ligated into the pGreenII 62-SK vector as effectors [41]. The resulting effector and reporter plasmids were co-infiltrated into tobacco leaves using the *A. tumefaciens* strain EHA105. The luciferase activity assay was determined according to the manufacturer’s instructions of the Dual Luciferase Assay Kit (Promega, Madison, WI, USA). All dual luciferase reporters measured were conducted by six replicates. The results were expressed by the LUC/REN ratio.

### 2.12. Yeast One-Hybrid Assay

Y1H assay was performed by using the Matchmaker Gold Yeast One-Hybrid System (Clontech, Fitchburg, WI, USA). The promoters of *MaBAM6/MaSEX4/MaMEX1* were ligated to pAbAi as the bait. The conducted plasmid was linearized and then transformed into the Y1H Gold strain. For the re-transformation assay, the full CDS of MaNAC67-like was transformed with the pGADT7-AD prey vector and then transferred into the aforementioned bait-reporter yeast strain. The interaction was analyzed according to the growth condition of the co-transformants on SD/-Leu medium with Aureobasidin A (AbA). All the primers used in present experiment are listed in Table S1.

### 2.13. Statistical Analysis

Three independent biological replicates were conducted for each treatment. Data are presented as the mean ± the standard deviation (SD). Charts were created using the SigmaPlot 12.0 software (Systat Software Inc, San Jose, CA, USA). Duncan’s multi-range test was conducted to compare the means among treatments. Least significant difference (LSD) was used to compare the significant effects at 5% level by using SPSS 16.0 software (SPSS, Inc., Chicago, IL, USA).

## 3. Results

### 3.1. Change in Ripening Parameters of Fruits during Storage and Fruit Ripening

**Figure 1:**
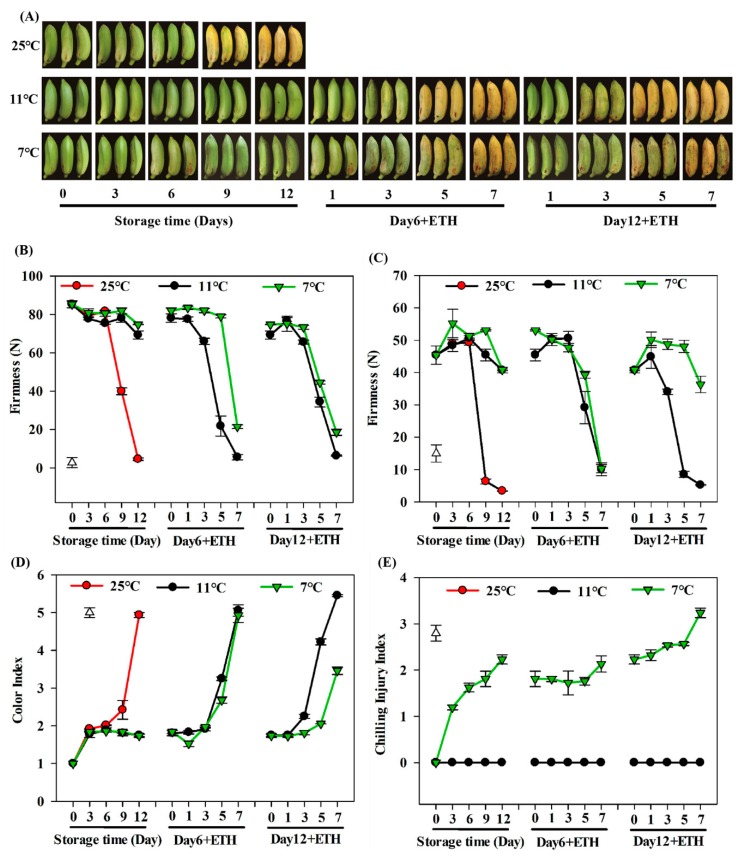
The ripening progress of Fenjiao fruit stored at 25 °C, 11 °C, and 7 °C for 6 and 12 days. (**A**) Picture of fruit during storage and ripening process; (**B**,**C**) fruit firmness changes in whole fruit (**B**) and pulp (**C**); (**D**,**E**) fruit color index (**D**) and chilling injury index (**E**) during banana fruit ripening. Fruit were stored at 25 °C, 11 °C, and 7 °C for 6 or 12 days, removed to 25 °C, and then treated with ethephon. The means of three biological replicates are presented, and the vertical bars show the standard error. Differences between various treatments were assessed by LSD at *p* = 0.05. ETH: ethephon.

To monitor changes in important ripening characteristics during fruit storage, major traits viz. fruit firmness, the color index, and the chilling index were determined for different storage conditions (7 °C, 11 °C, and 25 °C). Fruit ripened very quickly when placed at 25 °C, reaching a color index of 5 and peeling by 12 days after harvest (Figure 1A,D). Fruit firmness (both whole fruit firmness and pulp firmness) decreased rapidly after Day 6, reaching the lowest level of 0 N at Day 12 (Figure 1B,C). Ripening was estimated delayed when fruit were stored at 7 °C and 11 °C, when the fruit color index and fruit firmness remained at stable level for 12 days of storage at both temperatures (Figure 1A–D). After 12 days storage, fruit treated by ethephon and fruit pulp from the 7 °C storage were not able to completely soften at the end of shelf life, and were firmer than fruit receiving the 11 °C storage treatment (Figure 1C). Additionally, fruit stored at 7 °C were not able to completely turn yellow (Figure 1A,D). After 6 days of cold storage for fruit treated by ethephon, fruit was able to turn soft and yellow normally in both 7 °C and 11 °C storage, and no significant difference was observed at the end of storage (Figure 1A–D). For the 7 °C treatment, CI symptoms were observed in banana fruit by Day 6 and severe symptoms were observed by Day 12. For the 11 °C treatment, slight browning was observed after the ethephon treatment on Day 6 and Day 12 (Figure 1E). The severity of CI increased after 12 days at 7 °C by application of exogenous ethephon at 25 °C, where about 50% of peel browning was observed at the end of storage (Figure 1A,E).

**Figure 2:**
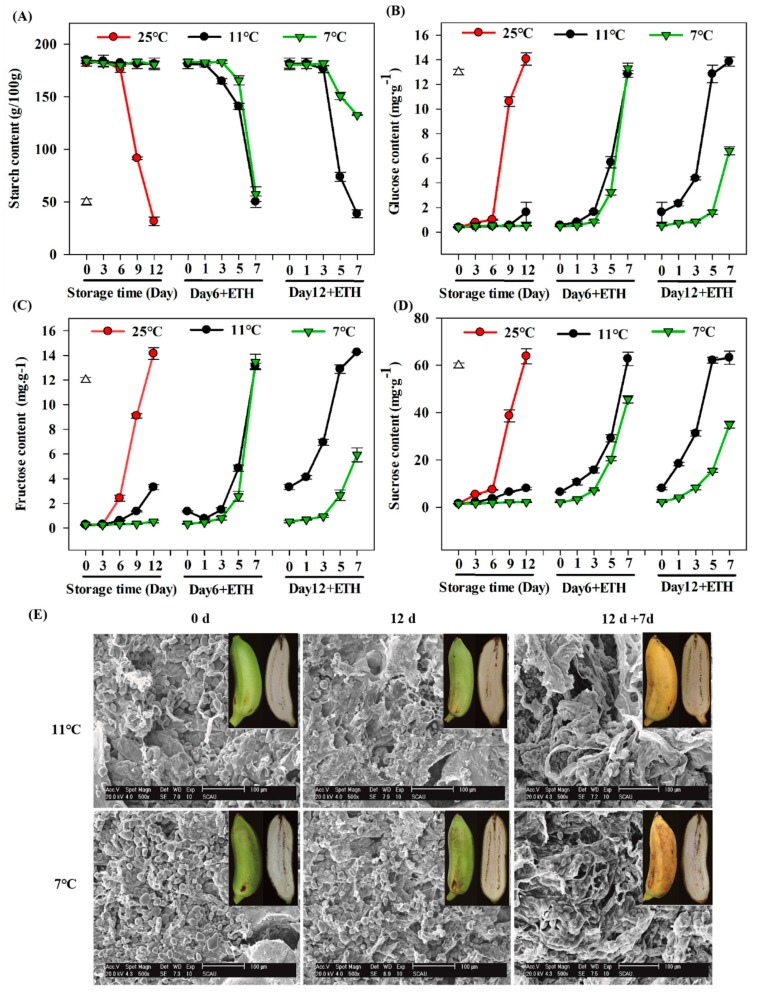
Changes in the content of: Starch (**A**,**E**), glucose (**B**), fructose (**C**), and sucrose (**D**) in banana fruit pulp during ripening. Fruit were stored at 25 °C, 11 °C, and 7 °C for 6 or 12 days, removed to 25 °C, and treated with ethephon. (**E**) Transmission electron microscopy pictures of Fenjiao fruit pulp close to core. Fruit pictured at harvest (0 d), after 12 days of cold storage, and at fully ripe stage following ethephon treatment.

The starch content for fruit stored at 25 °C decreased from Day 6 to Day 12, and a corresponding rapid increase in soluble sugar content of glucose, sucrose, and fructose was observed (Figure 2A–D). Both low-temperature treatments (7 °C and 11 °C) inhibited starch degradation and the starch and soluble sugar contents were stable during storage (Figure 2A–D). After 6 days of storage at 7 °C and 11 °C, and subsequent removal to 25 °C, the starch content decreased rapidly by exogenous ethephon application, and a corresponding rapid increase in soluble sugar content of glucose, sucrose, and fructose was observed (Figure 2A–D). However, by Day 12, the degradation of starch and the increase in soluble sugars were disturbed in fruit stored at 7 °C, and fruit contained starch that could not completely degrade even after exogenous ethylene application. This resulted in a lower soluble sugar content compared with the control. However, fruit stored at 11 °C had a normal starch degradation, and soluble sugars increased compared with the control (Figure 2A–D).

The microscopic effects of low-temperature treatments on the pulp tissues were determined by TEM observation [20]. There was a clear difference in the number of starch granules in the pulp of fruit stored at 7 °C and 11 °C during the fruit ripening process (Figure 2E). Fewer starch granules were observed at the ripe stage of fruit stored at 11 °C after ethephon treatment, but a greater number of starch granules were detected in fruit stored at 7 °C at the full ripening stage (Figure 2E). Therefore, the conversion of starch to sugar was impaired by 12 days of storage at 7 °C, and starch degradation was inhibited by chilling storage.

### 3.2. Expression Profiles of MaEBF1 and Starch Degradation-Related Genes

Based on the RNA-seq analysis, genes associated with starch degradation pathways and ethylene signal pathways were differentially expressed during Fenjiao fruit ripening and cold storage (Figures S1 and S2). Thirty-eight genes related to starch degradation were identified and characterized. The expression of *MaEBF1*, an important member in ethylene signal pathway, was repressed during fruit ripening, and induced by during cold storage.

**Figure 3:**
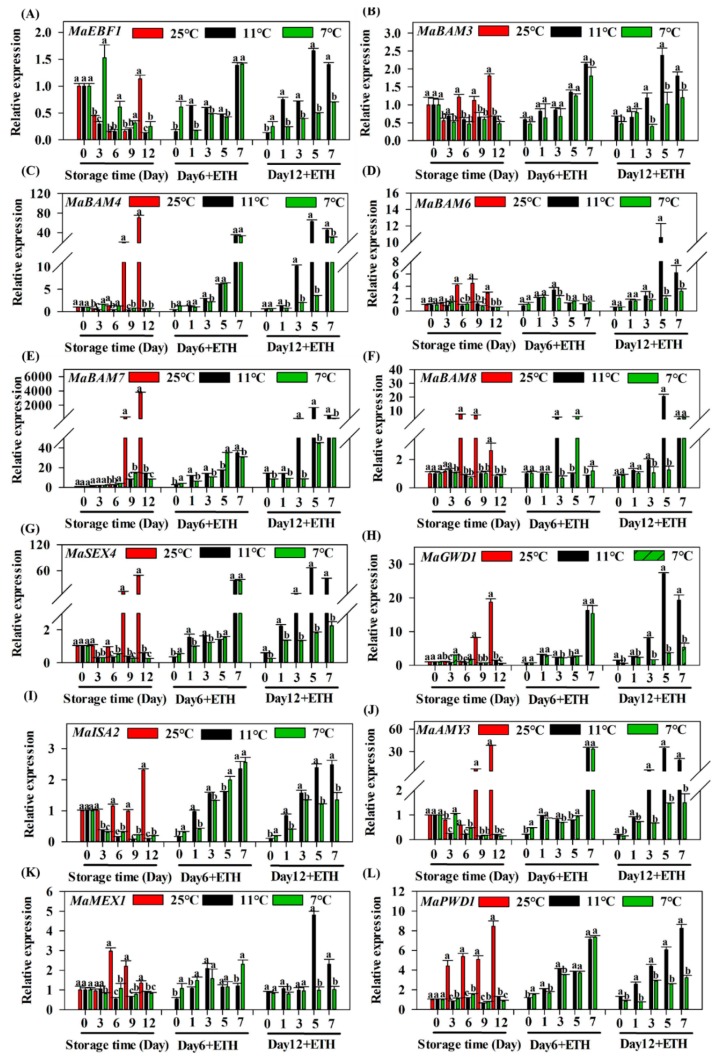
The expression profiles of genes related to starch degradation in Fenjiao fruit pulp under different storage conditions. The expression patterns of *MaEBF1* (**A**) and starch degradation genes (**B**–**L**) after different storage conditions and ripening process. The expression levels of each gene at different days are relative to that of 0 d (freshly harvested fruit), which was set as 1. Each value represents the means of biological triplicates (mean ± SD). Different letters above the bars indicate significant differences at the 5% level. Fruit were stored at 25 °C, 11 °C, and 7 °C for 6 or 12 days, removed to 25 °C, and treated with ethephon.

After 6 days of storage, the expression of *MaEBF1* was dramatically induced by ethephon treatment in fruit without CI, which were stored at both 7 °C and 11 °C (Figure 3A). After 12 days of storage, the expression of *MaEBF1* in fruit under 11 °C without CI increased rapidly by ethephon treatment with fruit ripening. Chilling stress inhibited the expression of *MaEBF1*, which was significantly lower in fruit stored at 7 °C than 11 °C storage (Figure 3A). As shown in Figures S1 and S2, genes *MaBAM3*, *MaBAM4*, *MaBAM6*, *MaBAM7*, *MaBAM8*, *MaAMY3*, *MaMEX1*, *MaISA2*, *MapGlcT2-2*, *MaGWD1*, *MaPWD1*, *MaLSF2*, and *MaSEX4* were involved in starch degradation and dramatically increased with fruit ripening and softening. The transcription levels of these genes dramatically increased with fruit ripening under normal conditions (25 °C), but were significantly repressed under cold storage (7 °C and 11 °C) (Figure 3B–L). After 6 days of cold storage, the expression of all genes increased with fruit ripening and no significant difference was observed between fruit subjected to 7 °C and 11 °C. However, after 12 days of storage, expression was significantly reduced following 7 °C storage, where fruit suffered serious chilling injury, and expression was significantly lower than the 11 °C treatment. Overall, these data indicate that the gene expression of *MaBAM3*, *MaBAM4*, *MaBAM6*, *MaBAM7*, *MaBAM8*, *MaAMY3*, *MaMEX1*, *MaISA2*, *MaGWD1*, *MaPWD1*, and *MaSEX4* was closely related to fruit softening and the starch content of the phenotype.

### 3.3. Protein Interactions Between MaEBF1 and MaNAC67-Like

**Figure 4:**
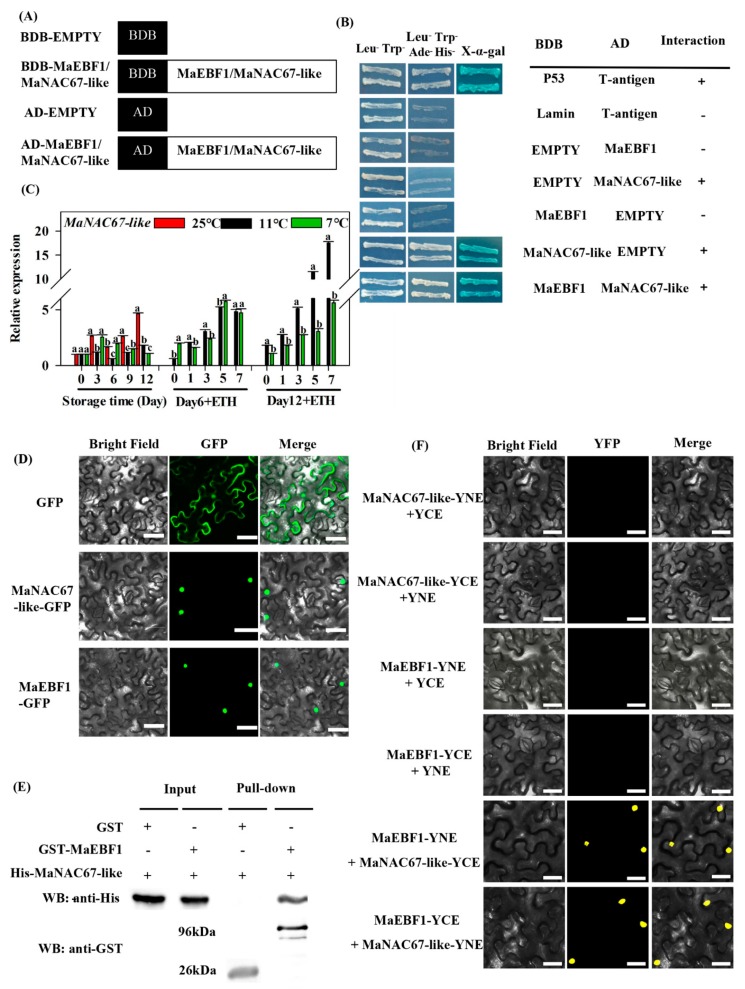
Interaction between MaEBF1 and MaNAC67-like proteins. (**A**,**B**) Y2H assay showing the interaction of MaEBF1 with MaNAC67-like. Diagram of the different constructs used in this experiment (**A**); the Y2H yeast strains were co-transformed with MaEBF1 + MaNAC67-like. Yeast cells grew on SD/-Leu-Trp-Ade-His in the presence of 125 μm Aureobasidin A, and blue plaques served as a positive interaction with chromogenic substrate X-α-gal (**B**); (**C**) the expression profile of MaNAC67-like during cold storage and ripening process; (**D**) the subcellular localization of MaEBF1 and MaNAC67-like proteins. (**E**) In vitro pull-down assay of the interaction between MaEBF1 with MaNAC67-like. (**F**) BiFC analysis of the interactions of MaEBF1 with MaNAC67-like in tobacco leaf epidermal cells. Bars, 50 μm.

The Y2H assays exploited to determine the transcription activation of MaEBF1 showed that MaEBF1 did not own transcriptional activation, and then MaEBF1 was chosen as a bait protein (Supplementary Figure S3). The cDNA library was assessed by electrophoresis, library fragment detection, and plate growth, which showed that it met the library standards (Clontech, 634901) (Supplementary Figure S4). MaNAC67-like was screened by the Y2H, which interacted with MaEBF1 (Figure 4A,B). Next, the full-length *MaEBF1* and *MaNAC67-like* CDS sequences were conducted to the GAL4-BD and GAL4-AD vectors, which were used to test their self-activation. The results indicated that pGBKT7-MaNAC67-like displayed self-activation activity, but others, including pGBKT7-EBF1, pGBADT7-MaEBF1, and pGBADT7-MaNAC67-like, did not. The Y2H assays showed that MaEBF1 interacted with MaNAC67-like (Figure 4A,B). Expression analysis showed that *MaNAC67-like* expression increased with fruit ripening. Both cold storage temperatures repressed the expression of *MaNAC67-like*, especially for fruit stored at CTS for 12 days. The expression of *MaNAC67-like* increased after ethephon treatment at Day 6 of storage at 7 °C and 11 °C, but there was no difference between 7 °C and 11 °C storage (Figure 4C). However, the expression of *MaNAC67-like* significantly reduced by Day 12 at 7 °C, when fruit suffered serious chilling injury. The expression of *MaNAC67-like* was significantly repressed by 7 °C and expression was lower than 11 °C.

The subcellular location analysis showed that both MaEBF1 and MaNAC67-like located in the nucleus (Figure 4D). The interaction between MaEBF1 and MaNAC67-like were then confirmed by BiFC and GST-pull down assays. The GST-pull down test showed that MaEBF1 could successfully pull down MaNAC67-like protein, indicating that they interacted with each other in vitro (Figure 4E). Additionally, BiFC fluorescence was observed in the nucleus of tobacco mesophyll cells by transiently transformation assay. Strong YFP fluorescent signals were detected in the nucleus when MaEBF1 was co-expressed with MaNAC67-like, whereas no YFP fluorescent signal was observed in MaEBF1-YFP^N^, MaEBF1-YFP^c^, MaNAC67-like-YFP^N^, and MaNAC67-like-YFP^c^ (Figure 4F). This shows that MaEBF1 interacted with MaNAC67-like in vivo (Figure 4F).

### 3.4. MaNAC67-Like Acts Cooperatively with MaEBF1 to Activate Starch Degradation-Related Genes

**Figure 5:**
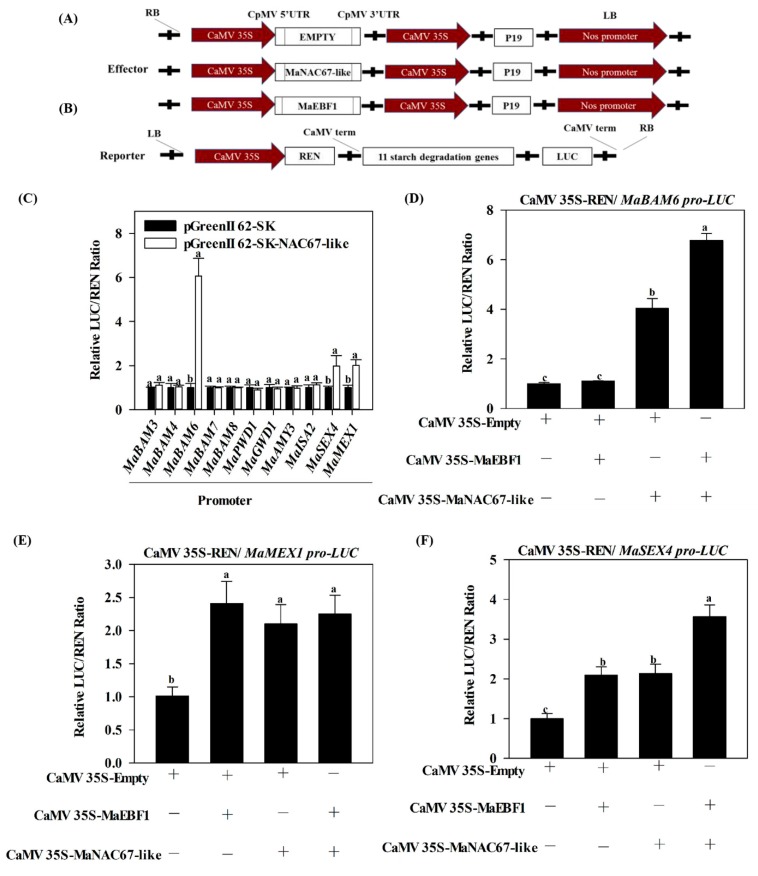
Transcriptional activation of starch degradation genes by MaEBF1 and MaNAC67-like in the transient expression system. The promoters of MaBAM6, MaMEX1, and MaSEX4 were ligated into a pGreenII 0800-LUC double-reporter vector (**A**), while MaNAC67-like was ligated into the pGreenII 62-SK vector as an effector (**B**). The transcriptional activity of MaNAC67-like on the starch degradation-related gene promoter of *MaBAM3*, *MaBAM4*, *MaBAM6*, *MaBAM7*, *MaBAM8*, *MaPWD1*, *MaGWD1*, *MaAMY3*, *MaISA2*, *MaSEX4*, and *MaMEX1* (**C**). The ratio of LUC to REN of the empty vector plus promoter vector was used as a calibrator (set as 1). (**D**–**F**) Transcriptional activity of MaEBF1, MaNAC67-like, and MaNAC67-like + MaEBF1 on the activities of promoter of *MaBAM6* (**D**), *MaMEX1* (**E**), and *MaSEX4* (**F**). Each value was calculated from the means of six biological replicates. The values represent the means ± SE.

Sequence analysis identified 11 selected genes (*MaBAM3*, *MaBAM4*, *MaBAM6*, *MaBAM7*, *MaBAM8*, *MaAMY3*, *MaMEX1*, *MaISA2*, *MaGWD1*, *MaPWD1*, and *MaSEX4*) with NACRS or NACBS elements in their promoters. The result suggests that MaNAC67-like might be direct target of these 11 selected genes. To determine whether MaNAC67-like and interactions with MaEBF1 affected the promoter activity of the 11 starch degradation genes, transient assays were performed using dual luciferase reporter assay. Among the 11 promoters tested, the activities of *MaBAM6*, *MaSEX4*, and *MaMEX1* promoters were dramatically induced by MaNAC67-like, with a higher LUC/REN ratio compared with that of the control (Figure 5C). MaNAC67-like had no effect on the promoter activities of other genes. When both MaNAC67-like and MaEBF1 were co-transformed, the LUC/REN ratio of *MaBAM6* and *MaSEX4* were more dramatically induced, and were much greater than the combined values observed for MaNAC67-like and MaEBF1 alone (Figure 5D,F). The interaction did not affect the promoter activity of *MaMEX1* (Figure 5E). These results indicate that MaNAC67-like regulates *MaBAM6*, *MaMEX1*, and *MaSEX4* individually, or that MaNAC67-like forms a protein complex with MaEBF1 to act cooperatively in the activation of *MaBAM6* and *MaSEX4* expression (Figure 5D,F).

**Figure 6:**
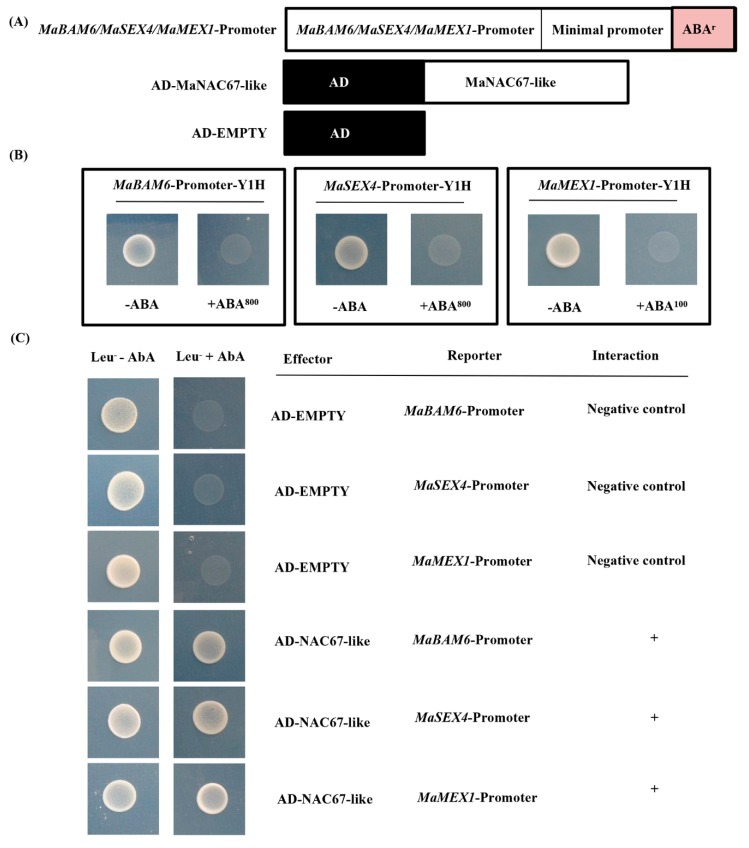
Y1H assay showing the ability of MaNAC67-like protein to bind promoters of *MaBAM6*, *MaSEX4*, and *MaMEX1*. (**A**) Diagram of the different constructs used in this experiment. (**B**) No basal expression of *MaBAM6*, *MaSEX4*, or *MaMEX1* promoters was observed in yeast grown on SD medium lacking Leu with 200 ng mL^−1^ AbA. (**C**) Yeast growth assays for determining the binding ability of MaNAC67-like to promoters of genes related to starch degradation. MaNAC67-like effector or empty (AD, negative control) plasmids were transformed into Y1H reporter strains. The interaction was assessed according to the growth condition of transformed yeast on SD medium lacking Leu but with AbA.

No basal activities of *MaBAM6*, *MaSEX4*, or *MaMEX1* promoters were observed in yeast (Figure 6B). Furthermore, the Y1H reporter strains that were transformed with plasmids carrying cassettes constitutively expressing the MaNAC67-like effector and yeast cells harboring *MaBAM6*, *MaSEX4*, or *MaMEX1* promoters grew well with AbA, indicating that MaNAC67-like can bind to *MaBAM6*, *MaMEX1*, and *MaSEX4* promoters in yeast (Figure 6C).

## 4. Discussion

During the fruit development of bananas, a large amount of starch is accumulated, which is converted into soluble sugar during the postharvest ripening stage [42]. The starch content takes up approximately 15–35% of the fresh weight of banana fruit [43]. Banana fruit softens and sweetens during ripening, which is not only due to cell wall hydrolases activities, but also to the starch-to-sugar metabolism process. Starch degrades to soluble sugars (sucrose, fructose, and glucose) and carbon substrate for aromatic volatile compounds production during fruit ripening [44]. Therefore, starch degradation is a critical process for fruit taste and flavor quality. Previous studies have also shown that the softening of banana fruit is closely related to starch degradation [34,45,46].

Softening and sweetening are two vital indicators of fruit ripening and quality in banana, and these processes partially result from the degradation of starch. During fruit ripening, starch degradation correlates well with a decline in pulp firmness [34]. The current study also supports the previous findings and shows that starch is rapidly degraded within banana fruit ripened at storage set to 25 °C and 11 °C (Figure 1 and Figure 2). However, fruit subjected to 7 °C for 12 days were prevented from softening completely even though fruit were treated by exogenous ethylene. The starch content of fruit stored at 7 °C was prevented from degrading completely and was clearly observed at the end of storage (Figure 2). A significantly lower soluble sugars content was also detected in fruit stored at 7 °C. Thus, the chilling-inhibited starch degradation severely impaired the taste and flavor of Fenjiao banana fruit.

Recently, 38 starch degradation-related genes were isolated and characterized in banana fruit and their expression was closely related to fruit ripening [36]. Among them, 27 genes were significantly up-regulated during banana fruit ripening and 18 starch degradation-associated enzymes were identified by iTRAQ-based proteomics experiments [36]. In the present study, the chilling temperature of 7 °C caused fruit softening problems, due to the fact that starch could not degrade completely. Based on the RNA-Seq analysis, the current study identified and characterized 38 genes related to starch degradation (Supplementary Figure S2). Several genes were significantly inhibited by the 7 °C treatment (Figure 3), along with an increase in total soluble sugars and decrease in starch content was inhibited during ripening (Figure 2). Thus, the current study identified that fruit pulp softening disorder caused by 7 °C storage is primarily due to the failure of starch degradation. Among these starch-associated genes, 11 were significantly increased with fruit ripening under 25 °C condition. Their expression increased more than 10-1000x during fruit ripening compared to day 0 (Figure 3), but severely repressed by cold storage, especially for those genes of *MaBAM4*, *MaBAM6*, *MaBAM7*, *MaSEX4*, *MaGWD1*, *MaAMY3*, and *MaPWD1*. These results indicate that these genes are important for starch degradation. Previous studies have also shown that the expression of these genes up-regulated with fruit ripening and induced by exogenous ethylene [36]. For example, in another starchy fruit, kiwifruit, the starch degradation and expression of *AdBAM3.1/3L/9*, *AdAMY1*, and *AdAGL3*, were significantly up-regulated during fruit ripening [47].

Although various evidence has shown that ethylene plays a critical role in the regulation of starch metabolism in bananas [36], there is no systematic analysis of the roles of ethylene response elements in regulating starch metabolism. Previous studies have analyzed the *cis* elements to diverse hormones in the promoter of 16 *MaBAM* genes, which showed that only *MaBAM3c* contained a single ethylene response (ERE) element in the promoter region [48]. Interestingly, ethylene plays a different role in starch degradation among banana cultivars, which show distinct ripening behaviors and different starch metabolism patterns. For example, dessert banana are normally sell in nature with high soluble sugar content, while plantain banana are usually as cooking bananas with high amounts of starch [34].

Transcription factors (TFs), including MADS-box, AP2/Ethylene Response Factor (ERF), NAC, SBP, and HD-ZIP, play critical roles in regulating fruit ripening by positively or negatively regulating ripening related genes [49,50]. However, the roles of these TFs in starch metabolism related to fruit quality during postharvest ripening has not been well studied. Recent studies have shown that a transcription factor, MabHLH6, activated the promoters of group starch degradation associated genes and may work as a positive regulator of starch degradation-associated genes in regulating fruit ripening [36]. NAC is one of the pivotal TF in plant. NAC is also an important actor in the fruit ripening process. In banana, MaNAC1/2 is involved in the ripening of banana fruits by interacting with ethylene signaling component MaEIL5 [26]. CpNAC1 positively regulates carotenoid biosynthesis by transcriptionally activating of CpPDS2/4 during fruit ripening in papaya [28]. SlNAC4 positive regulates fruit ripening by affecting carotenoid accumulation and ethylene synthesis in tomato, but SlNAC4 cannot be up-regulated by ethylene [30]. Furthermore, SlNAC4 interacts with both NOR and RIN proteins, and regulates fruit either in ethylene-dependent or in independent processes. SlNAC1 also inhibits fruit ripening via regulating carotenoid accumulation and ethylene synthesis, which interact with the regulatory domains of genes related ethylene and lycopene synthesis [51].

In the present work, the expression of *MaEBF1* was significantly induced by ETH treatment, which is similar to *EBF* genes in other fruits, such as *SlEBF1/2/3* in tomato [17,52] and *MaEBF2* in *Cavendish* banana fruit [53], indicating that *MaEBF1* may be an important player in Fenjiao banana fruit ripening. Additionally, the expression of *MaEBF1* was strongly reduced after 12 days at 7 °C, which may be closely related to the fruit ripening problem. MaNAC67-like interacted with MaEBF1 and its expression was strongly inhibited by 7 °C. The current study shows that MaNAC67-like was able to bind directly with the promoter of *MaBAM6*, *MaMEX1*, and *MaSEX4* in vivo (Figure 6), although the promoter binding ability of MaNAC67-like to these starch-related genes may need further experimental confirmation by electrophoretic mobility shift assay (EMSA) and chromatin immunoprecipitation (ChIP) assays. Furthermore, MaEBF1 cooperated with MaNAC67-like and strongly induced the expression of *MaBAM6* and *MaSEX4* (Figure 5). These results indicate that MaEBF1 and MaNAC67-like may function as a positive regulator of fruit starch metabolism. It was supposed that MaEBF1 interacts with MaNAC67-like and may be involved in starch degradation by regulating a group of starch degradation-related genes. However, further work, including targeted transgenic research, is necessary to fully unravel the biological function of MaEBF1 and MaNAC67-like in regulating starch degradation and fruit ripening.

## 5. Conclusions

Taken together, the current study shows that chilling-temperature storage caused ripening disorder in Fenjiao banana fruit, in which fruit could not fully soften, with higher starch content. *MaEBF1*, *MaNAC67-like*, and a series of starch degradation-related genes were identified and their expression was severely repressed by chilling-temperature storage. MaNAC67-like protein was able to physically bind to the promoter of genes associated with starch degradation, and the interaction with MaEBF1 further activated the promoters of some of these starch degradation-related genes. These results indicate that MaNAC67-like is a direct regulator of starch degradation and potential for involvement in regulating chilling-inhibited starch degradation by interacting with the ethylene signaling components in banana fruit. The present study expands our understanding of the intricate transcriptional regulatory network of starch degradation during fruit ripening, and will be useful for improving banana fruit chilling stress resistance.

## Supplementary Materials

The following are available online at https://www.mdpi.com/2218-273X/9/10/552/s1: Table S1. Summary of primers used in this study; Figure S1. RNA-Seq analysis showed that the genes in ethylene signal pathway were differentially expressed under three different storage and ripening conditions; Figure S2. Expression analysis of 38 starch degradation-associated genes in RNA-Seq analysis expressed under three different storage and ripening conditions; Figure S3. Transcription activation analysis of MaEBF1, MaEBF2, and MaERF1; Figure S4. cDNA library quality testing.
