# Supplementary material for: The Involvement of the Banana F-Box Protein MaEBF1 in Regulating Chilling-Inhibited Starch Degradation through Interaction with a MaNAC67-Like Protein
Source: Biomolecules. 2019 Sep 30;9(10):552. doi: 10.3390/biom9100552 (PMC6843822; doi:10.3390/biom9100552)
Supplement: Supplementary file 1 [file biomolecules-09-00552-s001.pdf]

Supplementary material

Article

The involvement of the banana F-box protein MaEBF1 in regulating chilling-inhibited starch degradation through interaction with a MaNAC67-like protein

Zunyang Song, Jiajia Qin, Qiuli Zheng, Xiaochun Ding, Weixin Chen, Wangjin Lu and Xueping Li*, Xiaoyang Zhu*

State Key Laboratory for Conservation and Utilization of Subtropical Agro-Bioresources/Guangdong Provincial Key Laboratory of Postharvest Science of Fruits and Vegetables, College of Horticulture, South China Agricultural University, Guangzhou, 510642, CHINA; songzunyang@163.com (Z.S); jia13424450489@163.com (J.Q.); zhengqiuli0102@163.com (Q.Z.); dingxiaochun111@163.com (X.D.);; wxchen@scau.edu.cn (W.C.); wjlu@scau.edu.cn (W.L.)

* Correspondence: lxp88@scau.edu.cn (X.L.) and xiaoyang_zhu@scau.edu.cn (X.Z.); Tel.: +86-20-38294892 (X.Z.)

Table S1. Summary of primers used in this study

Experiment	Gene	Forward primer (5'-3')
RT-qPCR	MaEBF1-F	CCTTCCTGACGAATGCCTCT
	MaEBF1-F	CAGAAGAACGGATGCTGCAC
	NAC-67-like-F	CGATGGATCAAGCGCAGATA
	NAC-67-like-R	GTGTGAGAGCACAGAGTAGTTC
	MaBAM3-F	TGCTGCGGACCACAGGCTT
	MaBAM3-R	GCTTTCCCAGGCGGTGTTCA
	MaBAM4-F	GCAAGAGGCATGGGGTGAAG
	MaBAM4-R	GCTCCTGATGAACTCGGTAAAC
	MaBAM6-F	TGATGGTGTCATGGTCGATTG
	MaBAM6-R	CCGCACGATCTGAAAGAGATG
	MaBAM7-F	GCCGACGACAGCATTGACCT
	MaBAM7-R	CAGCCATCTTCGAGTTCTTG
	MaBAM8-F	GATGGATCTTGGAACACACC
	MaBAM8-R	CGAACATGCGGAGTATTGGA
	MaGWD1-F	AGACTTCCCACAACATAGAG
	MaGWD1-R	AAGTGCCTGACAGATTACGA
	MaISA2-F	GCTGGAACTGTGGCGACGAA
	MaISA2-R	GTGGAGTAGCCGCACTCATC
	MaAMY3-F	AGGAACAGGCTCTGGGTATG
	MaAMY3-R	AGACTCAGTGGGTGGTGGTA
	MaMEX1-F	CCATATCAGTGCTCGTAGTGTC
	MaMEX1-R	CCGTAATGAAGTCCTCCCAAA
	MaPWD1-F	CAATAAGGCTGATGGGGATGA
	MaPWD1-R	AATGTCACTTTCTCCTGTCGG
	MaSEX4-F	GAAGAACTTACCTGAAGGACGC
Y2H	EBF1-Like-AD-F	ATGGCCATGGAGGCCAGTGAATTCATGGCGGCGCTCGTCAACT
	EBF1-Like-AD-R	TGCAGCTCGAGCTCGATGGATCCCCTAGGAAATGATATCGCACC
	NAC-67-like-AD-F	ATGGCCATGGAGGCCAGTGAATTCATGTCGAATCCTGCGTCGCTG
	NAC-67-like-AD-R	TGCAGCTCGAGCTCGATGGATCCCTCAGTGCAATCCCAGGTGGGAG
	EBF1-Like-BD-F	ATGGCCATGGAGGCCGAATTCATGGCGGCGCTCGTCAACT
	EBF1-Like-BD-R	TGCGGCCGCTGCAGGTCGACGCTAGGAAATGATATCGCACC
	NAC-67-like-BD-F	ATGGCCATGGAGGCCGAATTCATGTCGAATCCTGCGTCGCTG
	NAC-67-like-BD-R	TGCGGCCGCTGCAGGTCGACGTCAGTGCAATCCCAGGTGGGAG
GST-pull down	MaEBF1-GST-F	GATCTGGTTCCGCGTGGATCCATGGCGGCGCTCGTCAAC
	MaEBF1-GST-R	GTCACGATGCGGCCGCTCGAGCTAGGAAATGATATCGCACCAC
	NAC-67-like-his-F	CAGCAAATGGGTCGCGGATCCATGTCGAATCCTGCGTCG
	NAC-67-like-his-R	GTGGTGGTGGTGGTGCTCGAGGTGCAATCCCAGGTGGGAG
Subcellular location and BiFC	MaEBF1-GFP-F	CACCATGGCGGCGCTCGTCAACT
	MaEBF1-GFP-R	GGAAATGATATCGCACC
	NAC-67-like-GFP-F	CACCATGTCGAATCCTGCGTCGCTG
	NAC-67-like-GFP-R	GTGCAATCCCAGGTGGGAG
Transient expression assay	BAM3-0800-F	CTATAGGGCGAATTGGGTACCATTGCCTCCAGTACCTAACATTCAG

	BAM3-0800-R	TGTTTTTGGCGTCTTCCATGGCAT ACT GTC GGA GGC CGG CGA
	BAM4-0800-F	CTATAGGGCGAATTGGGTACCAGGGAATTATGAGAACATTTATATTG
	BAM4-0800-R	TGTTTTTGGCGTCTTCCATGGCCTCTCAAATTATAGAAGATGTAC
	BAM6-0800-F	CTATAGGGCGAATTGGGTACCATCATTTTTTGGTCGAAGGATGAG
	BAM6-0800-R	TGTTTTTGGCGTCTTCCATGGTTTCCGGGCAACAACGATC
	BAM7-0800-F	CTATAGGGCGAATTGGGTACCGATAAAGTGTTTGAAAGAACG
	BAM7-0800-R	TGTTTTTGGCGTCTTCCATGGAGCTGCAGCCAAAACGTG
	BAM8-0800-F	CTATAGGGCGAATTGGGTACCCCAGCCTTGGCTAGTCTT
	BAM8-0800-R	TGTTTTTGGCGTCTTCCATGGTCGAGTTGGTTCTCGAGG
	AMY3-0800-F	CTATAGGGCGAATTGGGTACCTCCTTCTGCGTTAGCCGTTG
	AMY3-0800-R	TGTTTTTGGCGTCTTCCATGGCGGCGAACGGTGGGGGAGAATC
	GWD1-0800-F	CTATAGGGCGAATTGGGTACCAATGACCCCATTAGATCGGATC
	GWD1-0800-R	TGTTTTTGGCGTCTTCCATGGACTTTCGTTGGAGGGACATCC
	ISA2-0800-F	CTATAGGGCGAATTGGGTACCTCTGTTAGCACTGAATAAGTGC
	ISA2-0800-R	TGTTTTTGGCGTCTTCCATGGGCAACAATGCTGGGTTGCCT
	MEX1-0800-F	CTATAGGGCGAATTGGGTACCTGGTGCTTGTGGTCCAGTG
	MEX1-0800-R	TGTTTTTGGCGTCTTCCATGGCTCAACTGTTCATCCATGGTGGA
	PWD1-0800-F	CTATAGGGCGAATTGGGTACCGTATGTGTCCCTGATTGTCAG
	PWD1-0800-R	TGTTTTTGGCGTCTTCCATGGCAGAGGTTAAGCTAAGAATCTAG
	SEX4-0800-F	CTATAGGGCGAATTGGGTACCTGGGCTTGTTCTGGGATTGAG
	SEX4-0800-R	TGTTTTTGGCGTCTTCCATGGCCACATAGGTGGGTGTGGCTG
	EBF1-SK-F	CGCTCTAGAACTAGTGGTACCATGGCGGCGCTCGTCAACT
	EBF1-SK-R	GATAAGCTTGATATCCCATGGCTAGGAAATGATATCGCACC
	NAC67-SK-F	CGCTCTAGAACTAGTGGTACCATGTCGAATCCTGCGTCGCTG
	NAC67-SK-R	GATAAGCTTGATATCCCATGGTCAGTGCAATCCCAGGTGGGAG
Y1H	BAM6-PAbAi-F	TTGAATTCGAGCTCGGTACCGCTCCCTCCGCTTCCTG
	BAM6-PAbAi-R	ATGCCTCGAGGTCGACTTTCCGGGCAACAACGATC
	MEX1-PAbAi-F	TTGAATTCGAGCTCGGTACCTCAATGACCATGTTAAGCTTTGAG
	MEX1-PAbAi-R	ATGCCTCGAGGTCGACGTGGCCGAGCTTGTGTTC
	SEX4- PAbAi -F	TTGAATTCGAGCTCGGTACCGAGATTTCTCGAAACAGTAGAAGTC
	SEX4-PAbAi-R	ATGCCTCGAGGTCGACGCACTCTTCTCCACATCACTAC
	NAC-67-like-AD-F	ATGGCCATGGAGGCCAGTGAATTCATGTCGAATCCTGCGTCGCTG
	NAC-67-like-AD-R	TGCAGCTCGAGCTCGATGGATCCCTCAGTGCAATCCCAGGTGGGAG

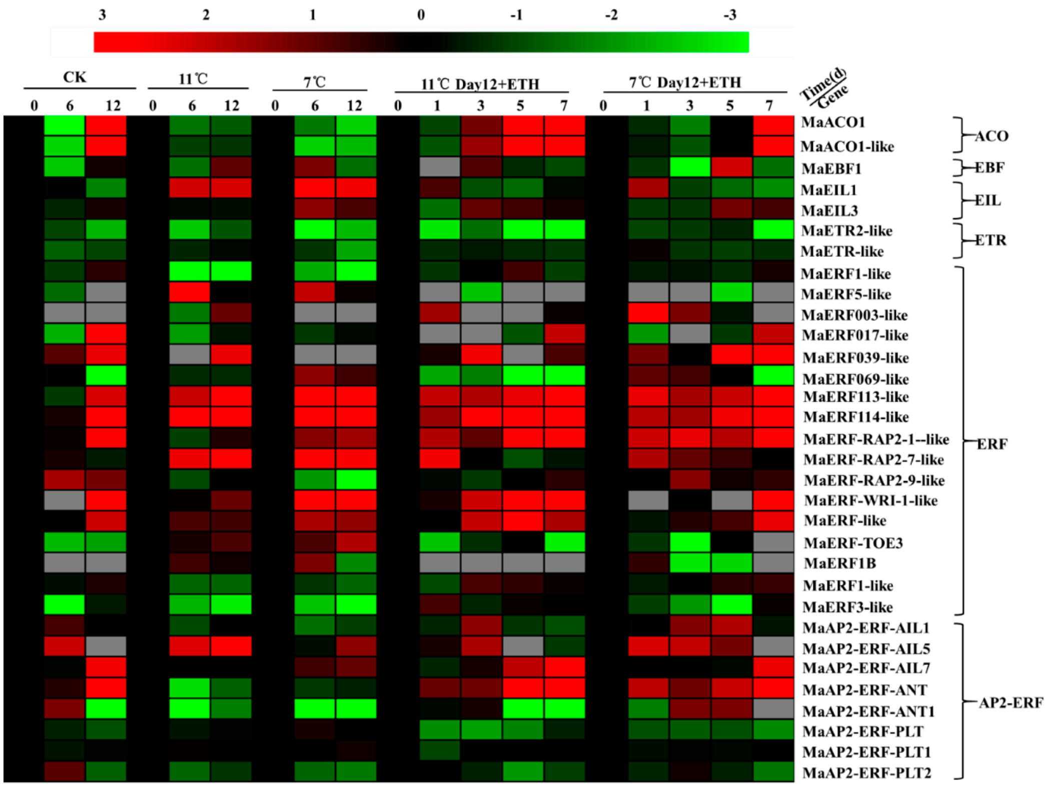

Figure S1. RNA-Seq analysis showed that the genes in ethylene signal pathway were differentially expressed under three different storage and ripening conditions: 25 °C, 11 °C, 7 °C. The expression image was generated using MeV software.

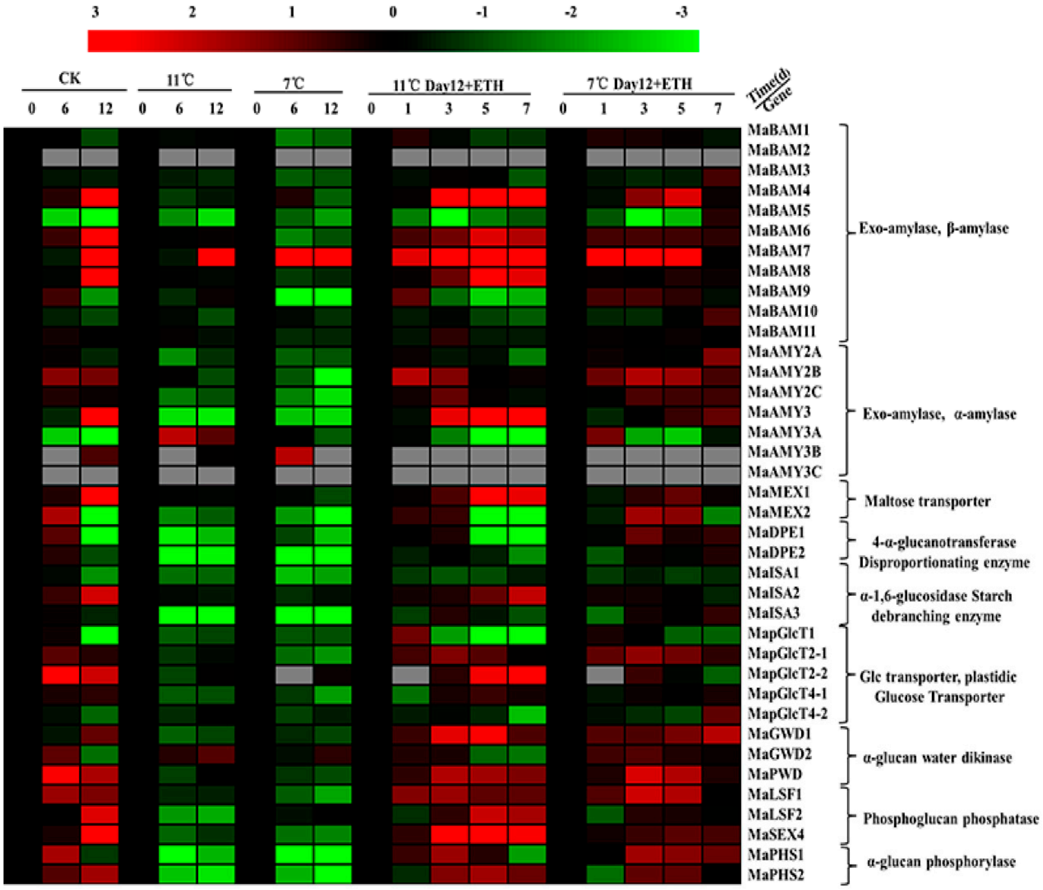

Figure S2. Expression analysis of 38 starch degradation-related genes in RNA-Seq analysis expressed under three different storage and ripening conditions: 25 °C, 11 °C, 7 °C. The expression image was generated using MeV software.

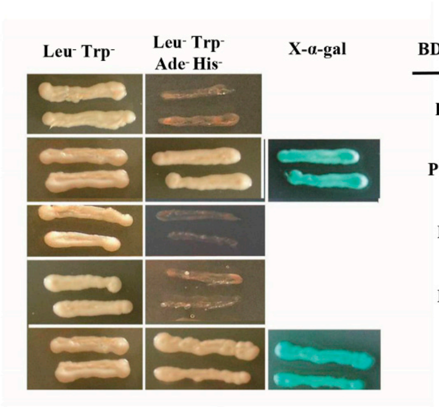

BDB (bait)	AD (prey)	Self-activation result
pGBKT7	-	-
PGBKT7-53	pGBKT7	+
MaEBF1	-	-
MaEBF2	-	-
MaERF1	-	+

Figure S3. Transcription activation analysis of MaEBF1, MaEBF2, and MaERF1.

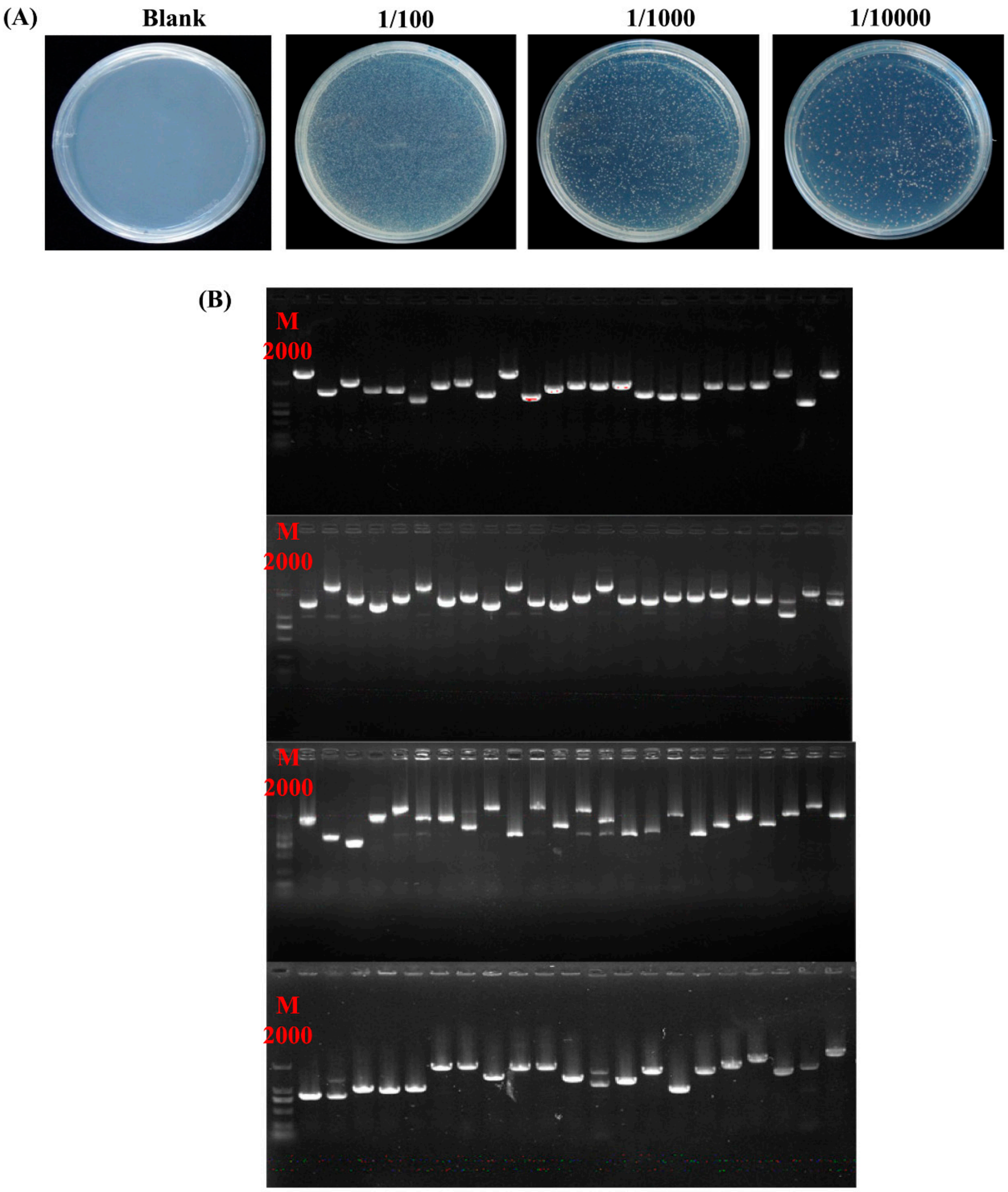

Figure S4. cDNA library quality testing. (A) The cDNA library was co-transformed into the Y187 strain with the pGADT7-rec vector. The yeast cells were grown on a synthetic medium lacking Leu and to diluted 10 times and100 times, respectively. (B) cDNA library inserted fragment quality testing.
